# Transcriptional rewiring in cancer driven by *POLR2A*/RPB1: mechanistic insights, non-coding RNA crosstalk, and therapeutic opportunities

**DOI:** 10.3389/fphar.2025.1745087

**Published:** 2025-12-19

**Authors:** Adrian Szczepaniak, Kinga Jaskuła, Marta Zielińska, Jakub Godlewski

**Affiliations:** 1 Department of NeuroOncology, Mossakowski Medical Research Institute, Polish Academy of Sciences, Warsaw, Poland; 2 Doctoral School of Translational Medicine, Centre of Postgraduate Medical Education, Warsaw, Poland; 3 Department of Experimental Genomics, Institute of Genetics and Animal Biotechnology, Polish Academy of Sciences, Jastrzębiec, Poland; 4 Department of Biochemistry, Faculty of Medicine, Medical University of Lodz, Lodz, Poland

**Keywords:** cancer, circular RNAs, non-coding RNAs, *POLR2A*, RPB1, viral infection

## Abstract

RNA polymerase II, together with its catalytic subunit RPB1 (encoded by *POLR2A*), forms the core of the eukaryotic transcriptional machinery that drives the synthesis of protein-coding and regulatory RNA transcripts. Accumulating evidence indicates that dysregulation of *POLR2A*/RPB1 is a critical driver of oncogenesis, promoting uncontrolled proliferation, evasion of apoptosis, and extensive transcriptional reprogramming across multiple malignancies, frequently affected by recurrent 17p deletions co-occurring with major tumor suppressor loss events. Such coordinated genomic alterations create transcriptional dependency that may be exploited therapeutically. Beyond its canonical role in transcription, *POLR2A*/RPB1 operates within an extensive regulatory network involving non-coding RNAs. Notably, circular RNAs derived from the *POLR2A* transcript have emerged as stable post-transcriptional regulators that modulate tumorigenic signaling pathways. In these roles, circular *POLR2A* isoforms promote proliferation, migration, and therapy resistance in glioblastoma and clear-cell renal cell carcinoma by acting as miRNA sponges or by scaffolding protein complexes that activate pathways such as ERK. These findings suggest that disturbances in *POLR2A* function reshape not only transcriptional output but also the broader non-coding RNA landscape, thereby reinforcing malignant phenotypes. Moreover, pharmacological agents such as triptolide further highlight transcription-dependent vulnerabilities by destabilizing RPB1, offering promising therapeutic opportunities, particularly in drug-resistant cancers. Collectively, *POLR2A*/RPB1 emerges as a central node linking transcriptional control, noncoding RNA biogenesis, and oncogenic signaling, positioning it as a compelling candidate for biomarker development and targeted therapeutic intervention.

## 1 Introduction

The transcriptional machinery of human cells is a finely tuned system that converts the genetic information stored in DNA into functional RNA molecules. At the center of this process is RNA polymerase II (Pol II), the enzyme complex responsible for transcribing all protein-coding genes as well as many non-coding RNA species, including small nuclear RNAs (snRNAs) and microRNAs (miRNAs), which are essential for RNA processing and post-transcriptional regulation (Vlaming et al., 2022). Pol II is composed of 12 subunits, with RPB1, encoded by *POLR2A*, serving as its most significant and catalytically active core component (Schier and Taatjes, 2020). Proper assembly of these subunits yields a highly efficient transcriptional machine capable of accurately converting the genomic template into messenger RNA. Throughout the transcription cycle - initiation, elongation, and termination - Pol II forms dynamic interactions with transcription factors, mediator complexes, and chromatin regulators that together ensure transcriptional fidelity (Muniz et al., 2021). Because of this central role, Pol II is essential for cell survival and homeostasis.

A hallmark of RPB1 is its C-terminal domain (CTD), composed of multiple tandem repeats of the heptapeptide motif YSPTSPS. Reversible phosphorylation of these repeats coordinates transitions between transcriptional stages and recruits RNA-processing factors required for 5′-capping, splicing, and polyadenylation. These CTD modifications also integrate Pol II function with chromatin remodeling, DNA damage repair, and epigenetic signaling (Yague-Sanz et al., 2023). Comparative studies revealed that RPB1 is highly conserved across eukaryotes; notably, yeast RPB1 can be replaced by its murine ortholog without loss of enzymatic function, underscoring its fundamental role in transcriptional regulation and cellular integrity (Bernecky et al., 2016).

Given its essential function, disruption of *POLR2A*/RPB1 has widespread consequences. Defects in the complex trigger global transcriptional dysregulation, impaired stress responses, and genomic instability, contributing to various diseases, including cancer (Liu et al., 2015; Li et al., 2018; Xu et al., 2019) and neurodevelopmental disorders (Hansen et al., 2021; Evans et al., 2022). In cancer, *POLR2A* is frequently overexpressed across tumor types, including breast (Xu et al., 2019), lung (Zeng et al., 2021), colon carcinomas (Liu et al., 2015), and glioblastomas (Wei et al., 2019). Elevated *POLR2A* levels enhance transcription of genes that support cell proliferation, survival, and metastasis, thereby promoting tumorigenesis. In addition, mutations or aberrations in the regulatory mechanisms–e.g., aberrant CTD phosphorylation–can further destabilize gene expression programs and accelerate cancer progression (Yi et al., 2016).

This mini-review summarizes current knowledge on the role of *POLR2A*/RPB1 in cancer biology, emphasizing its function as a central Pol II subunit. Integrating both experimental and computational evidence, we discuss how *POLR2A* dysregulation contributes to tumorigenesis and evaluate its potential as a prognostic biomarker and therapeutic target. We also highlight an emerging perspective on the interplay between *POLR2A*/RPB1 and non-coding RNAs, particularly circular RNAs derived from the linear *POLR2A*. These circRNAs may act as stable post-transcriptional regulators that link transcriptional imbalance to oncogenic signaling, opening new avenues for biomarker discovery and targeted therapy.

## 2 Distinct *POLR2A*/RPB1 expression patterns in various cancers

**FIGURE 1:**
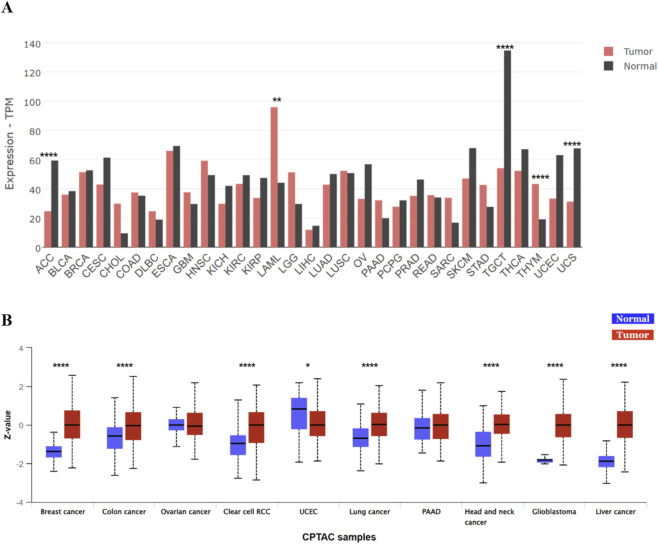
**(A)** Expression of *POLR2A* across various cancers (source: GEPIA2 analyzer). **(B)** Expression of RPB1 across various cancers (source: UALCAN analyzer). Z-values represent the standard deviation from the median across samples for each cancer type. TPM–Transcripts Per Million; ACC–Adrenocortical carcinoma; BLCA–Bladder Urothelial Carcinoma; BRCA–Breast invasive carcinoma; CESC–Cervical squamous cell carcinoma and endocervical adenocarcinoma; CHOL–Cholangio carcinoma; COAD–Colon adenocarcinoma; DLBC–Lymphoid Neoplasm Diffuse Large B-cell Lymphoma; ESCA–Esophageal carcinoma; GBM–Glioblastoma multiforme; HNSC–Head and Neck squamous cell carcinoma; KICH–Kidney Chromophobe; KIRC–Kidney renal clear cell carcinoma; KIRP–Kidney renal papillary cell carcinoma; LAML–Acute Myeloid Leukemia; LGG–Brain Lower Grade Glioma; LIHC–Liver hepatocellular carcinoma; LUAD–Lung adenocarcinoma; LUSC–Lung squamous cell carcinoma; OV–Ovarian serous cystadenocarcinoma; PAAD–Pancreatic adenocarcinoma; PCPG–Pheochromocytoma and Paraganglioma; PRAD–Prostate adenocarcinoma; RCC–Reanl Cell Carcinoma; READ–Rectum adenocarcinoma; SARC–Sarcoma; SKCM–Skin Cutaneous Melanoma; STAD–Stomach adenocarcinoma; TGCT–Testicular Germ Cell Tumors; THCA–Thyroid carcinoma; THYM–Thymoma; UCEC–Uterine Corpus Endometrial Carcinoma; UCS–Uterine Carcinosarcoma. * adjusted p-value <0.05; ** adjusted p-value <0.01; **** adjusted p-value <0.0001.

A comparative analysis of *POLR2A* expression across multiple cancer types, conducted using the GEPIA2 platform (Tang et al., 2019), revealed substantial upregulation of *POLR2A* in malignancies such as acute myeloid leukemia and thymoma. In contrast, strong downregulation was detected in adrenocortical carcinoma, testicular germ cell tumors, and uterine carcinosarcoma (Figure 1A). To complement these transcript-level observations, protein abundance data from the Clinical Proteomic Tumor Analysis Consortium (CPTAC) (Li et al., 2023) were analyzed via the UALCAN platform (Chandrashekar et al., 2022) to assess RPB1 expression (Figure 1B). Notably, changes in *POLR2A* mRNA levels did not always correspond to changes in RPB1 protein levels. For instance, in breast cancer and glioblastoma (GBM), *POLR2A* levels showed only minor variation, whereas pronounced differences emerged at the protein level (Figures 1A,B). These inconsistencies point to the influence of post-transcriptional regulation, such as changes in mRNA stability, translation efficiency, or protein turnover, that can modulate RPB1 abundance independently of *POLR2A* transcription.

Taken together, these results demonstrate that *POLR2A*/RPB1 is broadly dysregulated across cancers, with considerable variability between tumor types. This heterogeneity underscores the importance of further investigation into the regulatory mechanisms governing their expression and the potential of these molecules as biomarkers or therapeutic targets.

## 3 Genomic proximity of *POLR2A* to key tumor suppressor genes: implications for cancer biology and therapy

**FIGURE 2:**
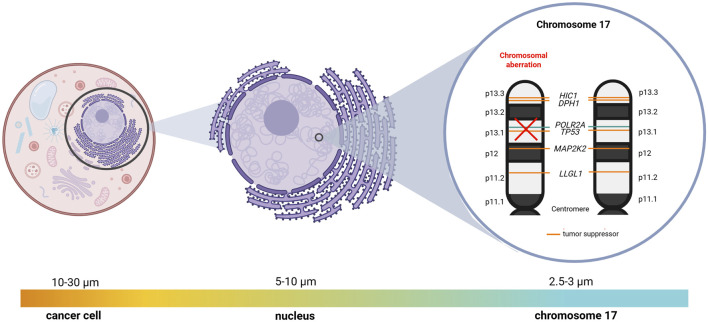
Diagram describing co-deletions in *POLR2A* and *TP53* genes (Created with BioRender.com).

*POLR2A,* located on chromosome 17p13.1, lies in proximity to the primary tumor suppressor hub - *TP53* (Figure 2). Because hemizygous *TP53* deletion is common across many cancer types (Yu et al., 2019; van Kampen et al., 2025), *POLR2A* is frequently co-deleted, resulting in haploinsufficiency of both genes and creating a potential therapeutic vulnerability (Muller et al., 2012; Nijhawan et al., 2012). This genomic relationship has led to the concept of selectively targeting the remaining *POLR2A* allele to impair tumor cell survival while sparing normal tissues.

In triple-negative breast cancer (TNBC), loss of one *POLR2A* copy often accompanies *TP53* deletion (Xu et al., 2019). Delivery of *POLR2A*-targeting siRNA via pH-activated nanoparticles effectively suppressed *POLR2A*-deficient tumors *in vivo* without systemic toxicity. These nanoparticles accumulated in both *POLR2A* ^neutral^ and *POLR2A* ^loss^ tumors, but inhibition of *POLR2A* significantly reduced tumor growth in heterozygous *POLR2A* ^loss^ tumors, still avoiding systemic side effects (Xu et al., 2019). A similar pattern is observed in colorectal cancer (CRC), where nearly all TP53-hemizygous loss cases show concurrent *POLR2A* deletion (Liu et al., 2015). In these models, inhibition of *POLR2A* by α-amanitin or specific siRNA markedly reduced proliferation and tumor growth in *POLR2A* ^loss^ models, independently of p53 status, suggesting a p53-independent therapeutic mechanism (Liu et al., 2015). These findings indicate that therapeutic targeting of *POLR2A* may represent a broadly effective, p53-independent strategy for tumors harboring TP53 deletions.

In castration-resistant prostate cancer (CRPC), 17p deletions occur in approximately 63% of metastatic cases (Li et al., 2018). In this context, RPB1 interacts with the E3 ligase RBX1, which enhances its activity via K63-linked ubiquitination, thereby augmenting RNAP2-mediated mRNA synthesis. Supporting the relevance of this mechanism, TCGA prostate cancer data show an inverse correlation between RBX1 and *POLR2A* expression (R = −0.45), suggesting that the loss of *POLR2A* in cells with deletion of the 17p chromosomal region may be functionally compensated by RBX1 upregulation (Li et al., 2018). Consequently, combined inhibition of RNAP2 and RBX1 markedly suppresses CRPC growth through a synergistic effect.

Beyond chromosomal deletions, somatic *POLR2A* mutations have also been identified in a cohort of 775 meningioma patients lacking known driver alterations; recurrent mutations such as p.Gln403Lys or a deletion of the p.Leu438_His439 region were detected (Clark et al., 2016). These tumors were genomically stable and had a low mutation burden, comparable to meningomas driven by established genes. Functionally, *POLR2A* mutations disrupted the expression of key meningeal identity genes (WNT6, ZIC1/ZIC4), defining a distinct and clinically unique meningioma subgroup (Clark et al., 2016). Collectively, these findings reinforce the central importance of *POLR2A* in transcriptional regulation and highlight its therapeutic vulnerability, particularly in cancers with 17p deletions or *POLR2A* mutations.

## 4 Diverse cellular pathways underlying *POLR2A*-driven oncogenesis

*POLR2A* is markedly upregulated in gastric cancer (GC) tissues compared with adjacent non-tumor tissue, and its elevated expression correlates with larger tumor size, deeper invasion, and poorer prognosis (Jiang et al., 2021). Functional studies revealed that *POLR2A* knockdown in *POLR2A* ^high^ cells markedly reduced viability. In contrast, its overexpression in *POLR2A* ^low^ cells enhanced viability and inhibited apoptosis by upregulating BCL2 and PARP, supporting its role in GC growth both *in vitro* and *in vivo* (Jiang et al., 2021). In lung adenocarcinoma, *POLR2A* expression strongly correlates with *BCAR1* (breast cancer antiestrogen resistance protein 1) overexpression and with unfavorable clinical outcomes (Mao et al., 2020). *BCAR1* knockout markedly suppressed cell proliferation and colony formation and, significantly, led to a substantial reduction in RPB1 levels. This suggests a regulatory interaction between BCAR1 and RPB1 that may contribute to the aggressive behavior of lung adenocarcinoma (Mao et al., 2020). In acute myeloid leukemia (LAML), both *POLR2A* and its catalytic subunit RPB1 are aberrantly activated, with RPB1 levels positively correlating with tumor burden and poor outcome (Yu et al., 2019). Global transcriptomic profiling showed that *POLR2A* knockout markedly suppressed oncogenic and antiapoptotic genes, including *MYC*, *RUNX2*, *MEIS1*, *CDC25A*, and *BCL-2*, leading to impaired proliferation and a significant reduction in tumor cell populations at 7 and 14 days after implantation *in vivo* (Yu et al., 2019).

Beyond changes in expression levels, the subcellular localization of RPB1 also appears to shape tumor behavior and therapy response. Although RPB1 normally resides in the nucleus, cancer cells can form cytoplasmic RPB1 aggregates, particularly in therapy-resistant breast and renal tumors (Nagy-Mikó et al., 2023). These aggregates, absent in normal tissue, likely reflect dysregulated translation as a compensatory response to genotoxic stress. Importantly, their presence in pretreatment biopsies may serve as a predictive marker of chemotherapy resistance (Nagy-Mikó et al., 2023).

Taken together, these findings show that *POLR2A* and RPB1 contribute to oncogenesis through multiple mechanisms, including regulation of apoptosis, cell cycle progression, and transcription of oncogenic programs. Moreover, both their expression patterns and subcellular localization hold promise as prognostic indicators and therapeutic targets across diverse cancer types.

## 5 Targeting RPB1 in cancer therapy: transcriptional vulnerabilities as a therapeutic opportunity

Triptolide, a diterpenoid compound originally isolated from *Tripterygium wilfordii*, has attracted considerable attention for its anticancer activity. It acts on multiple cellular pathways, including transcriptional regulation, apoptosis, cell cycle progression, oxidative stress responses, and autophagy (Bao et al., 2024; Feng et al., 2024). Mechanistic studies by Manzo et al. (Manzo et al., 2012) showed that triptolide selectively induces phosphorylation of the CTD at Ser-5 as an early event, followed by a progressive decrease in total RPB1 levels. Triptolide also caused promoter-proximal stalling of RNA polymerase II and reduced chromatin-bound Pol II, highlighting a direct impact on transcriptional initiation (Manzo et al., 2012). The importance of RPB1 degradation for drug sensitivity was further demonstrated in multidrug-resistant cancer models, where RPB1 was critical for mediating triptolide’s cytotoxic effects (Yi et al., 2016). CDK7-dependent phosphorylation of RPB1 at Thr170 promoted its degradation, while treatment with the selective CDK7 inhibitor BS-181 partially rescued cell viability (Yi et al., 2016). Together, these findings establish RPB1 destabilization as a hallmark of triptolide’s mechanism of action and suggest that RPB1 itself represents a promising therapeutic vulnerability, particularly in cancers resistant to conventional chemotherapies.

## 6 Targeting *POLR2A*/RPB1: viral strategies for transcriptional dominance

Many viruses that rely on host transcription have evolved sophisticated mechanisms to manipulate or disable *POLR2A*/RPB1 to seize control of cellular gene expression. By targeting this essential enzyme, viruses establish transcriptional dominance, boosting viral RNA synthesis while suppressing host antiviral defenses.

RNA viruses such as influenza and bunyaviruses hijack Pol II-derived capped RNA fragments to initiate their own mRNA synthesis. This strategy not only ensures efficient translation of viral transcripts but also helps the virus evade innate immune detection (Ding and Summers, 2022; Xu M. et al., 2022). In contrast, nuclear DNA viruses, including herpesviruses and adenoviruses, reprogram Pol II activity more directly. They modify CTD phosphorylation patterns to suppress host transcription while maintaining robust viral gene expression selectively (Zaborowska et al., 2014; Isa et al., 2021; Huang et al., 2022; Whisnant et al., 2024).

Cytoplasmic viruses, such as alphaviruses, bunyaviruses, and coronaviruses, often deploy a more aggressive approach. They trigger ubiquitin-proteasome-mediated degradation of RPB1, causing a rapid, global shutdown of host mRNA synthesis (Akhrymuk et al., 2012; Schoen et al., 2020; Hardy et al., 2024). Despite their diverse replication strategies, these viruses converge on a common principle: disabling RPB1, either through functional inactivation or degradation, is an efficient way to silence host defenses and monopolize the transcriptional machinery.

This repeated viral targeting of *POLR2A*/RPB1 highlights its central importance in transcriptional fidelity and cellular stress resilience. Intriguingly, cancer cells display analogous dependencies, exploiting *POLR2A*/RPB1 dysregulation to sustain aberrant transcriptional programs and survive genotoxic stress. Recognizing this functional overlap between viral infection and tumor biology opens new therapeutic opportunities: oncolytic viruses and transcription-targeted agents may be strategically leveraged to disrupt tumor-specific vulnerabilities driven by the *POLR2A*/RPB1 axis.

## 7 Deciphering the regulatory nexus: crosstalk between *POLR2A* and non-coding RNAs

Growing evidence indicates that non-coding RNAs (ncRNAs) – once regarded as the “dark matter” of the genome–are key regulators of cancer development and progression (Guzel et al., 2019; Duan et al., 2023). Although they do not encode proteins, ncRNAs are highly abundant: over 98% of the human genome is non-coding, and roughly 93% of these sequences are actively transcribed (ENCODE Project Consortium, 2012; Wang, 2022). The ncRNA family comprises several subclasses, including miRNAs, long non-coding RNAs (lncRNAs), and the more recently identified circular RNAs (circRNAs) (Szczepaniak et al., 2023). Emerging studies link *POLR2A* to multiple aspects of ncRNA biology, showing that it regulates lncRNA expression and also serves as a transcriptional template for circRNA biogenesis, suggesting a broad influence on the non-coding transcriptome.

In the canonical model, lncRNAs are transcribed by Pol II and regulated by various protein factors (Statello et al., 2021; Mattick et al., 2023). However, large-scale analyses have proposed additional, noncanonical modes of lncRNA transcription, potentially involving unknown regulators or lncRNA-lncRNA interactions (Wang, 2022). Intriguingly, lncRNAs interact with molecular effectors–such as transcription factors, histone modifications, or other regulatory proteins–in patterns that are noticeably different from those seen with protein-coding genes. Only ∼12% of lncRNA promoter regions (defined as ±5 kb around the transcription start site) are predicted to bind RPB1 (Wang, 2022), challenging the long-standing assumption that Pol II is the primary driver of all lncRNA transcription. Several studies have specifically implicated *POLR2A*-associated lncRNAs in cancer. In ovarian cancer, Guo et al., 2019) demonstrated that the signature of *POLR2A*-associated lncRNAs is more sensitive and specific for predicting survival than current clinical and molecular markers. Their findings showed that the *POLR2A*-lncRNA signature effectively stratified patients into high- and low-risk groups based on overall, progression-free, and disease-free survival. Furthermore, the signature effectively divided patients into distinct risk subgroups across various clinical parameters, including age, tumor grade, stage, and residual tumor diameter (Guo et al., 2019). These findings highlight the potential of *POLR2A*-linked lncRNAs as robust prognostic biomarkers and possible therapeutic targets.

Beyond its regulatory role in linear ncRNA expression, *POLR2A* also serves as a template for circRNA production (Chen et al., 2022; Xu Z. et al., 2022). CircRNAs are generated through a unique back-splicing process, in which a downstream 5′splice site covalently joins an upstream 3′splice site, developing a closed-loop structure devoid of free ends. This circular structure confers exceptional stability and resistance to exonuclease degradation (Yu and Kuo, 2019). Functionally, circRNAs act as miRNA sponges, serve as scaffolds for protein interactions, modulate transcription, and, in some cases, can even be translated (Verduci et al., 2021). Increasingly, circRNAs have been linked to oncogenic signaling and are emerging as promising biomarkers and therapeutic targets (Bronisz et al., 2020; Shi et al., 2024).

According to circBase, at least 77 *POLR2A*-derived circRNAs have been identified (Table 1) (Glažar et al., 2014). Among them, circ*POLR2A* (*hsa-POLR2A_0005*) is the most extensively studied. This circRNA is strongly upregulated in clear cell renal cell carcinoma (ccRCC), including metastatic lesions, and promotes proliferation, migration, invasion, and angiogenesis while inhibiting apoptosis (Xu Z. et al., 2022). Mechanistically, *hsa-POLR2A_0005* forms a ternary complex with UBE3C and PEBP1, thereby enhancing PEBP1 ubiquitination and degradation, activating the ERK signaling pathway, and driving tumor progression. In addition, m^6^A modification of *hsa-POLR2A_0005* modulates its stability and function, further influencing ccRCC pathogenesis. Targeting the *hsa-POLR2A_0005/*UBE3C/PEBP1 axis, therefore, represents a potential therapeutic approach for this aggressive malignancy (Xu Z. et al., 2022). *hsa-POLR2A_0005* is also upregulated in GBM (Chen et al., 2022), where it promotes proliferation and inhibits apoptosis. Subcellular fractionation revealed that hsa-POLR2A_0005 is predominantly cytoplasmic and acts as a sponge for miR-2113. By sequestering miR-2113, *hsa-POLR2A_0005* upregulates POU3F2, thereby activating SOX9 transcription through direct interaction with its promoter. These findings reveal a novel mechanistic pathway driving GBM progression, suggesting that targeting the *hsa-POLR2A_0005* miR-2113/POU3F2/*SOX9* axis could offer valuable therapeutic opportunities (Chen et al., 2022). Although hsa-*POLR2A*_0005 is the best characterized, many other circRNAs derived from the *POLR2A* locus likely participate in transcriptional or post-transcriptional regulation and remain to be explored.

**TABLE 1:** List of circRNAs derived from *POLR2A* deposited in the circAtlas database.

circAtlas ID	Position	Strand	circRNA type	Multiple conservation score	Spliced length	Algorithm
*hsa-POLR2A_0001*	chr17:7495941|7499491	+	Exonic	3.0303	1578	find_circ, CIRI2,DCC,CIRCexplorer2
*hsa-POLR2A_0002*	chr17:7495941|7497045	+	Exonic	3.0303	726	find_circ, CIRI2,DCC,CIRCexplorer2
*hsa-POLR2A_0003*	chr17:7511406|7511588	+	Exonic	3.0303	183	find_circ, CIRI2,DCC,CIRCexplorer2
*hsa-POLR2A_0004*	chr17:7508944|7509190	+	Exonic	2.0303	247	find_circ, CIRI2,CIRCexplorer2
*hsa-POLR2A_0005*	chr17:7499039|7499491	+	Exonic	2.90909	336	find_circ, CIRI2,DCC,CIRCexplorer2
*hsa-POLR2A_0006*	chr17:7499039|7503791	+	Exonic	2.21212	131	find_circ, CIRI2,DCC,CIRCexplorer2
*hsa-POLR2A_0007*	chr17:7499039|7508475	+	Exonic	1.0303	Unknown	find_circ, CIRI2,DCC,CIRCexplorer2
*hsa-POLR2A_0008*	chr17:7503121|7512344	+	Exonic	1.06061	187	find_circ, CIRI2,DCC,CIRCexplorer2
*hsa-POLR2A_0009*	chr17:7501289|7501600	+	Exonic	1.0303	170	CIRI2
*hsa-POLR2A_0010*	chr17:7499039|7509632	+	Exonic	1.09091	131	find_circ, CIRI2,DCC,CIRCexplorer2
*hsa-POLR2A_0011*	chr17:7511215|7511588	+	Exonic	2.0303	288	find_circ, CIRI2,DCC,CIRCexplorer2
*hsa-POLR2A_0012*	chr17:7503121|7503791	+	Exonic	1.06061	484	find_circ, CIRI2,DCC,CIRCexplorer2
*hsa-POLR2A_0013*	chr17:7497711|7500686	+	Exonic	1.0303	544	CIRI2,DCC
*hsa-POLR2A_0014*	chr17:7503121|7511588	+	Exonic	1.09091	183	find_circ, CIRI2,DCC,CIRCexplorer2
*hsa-POLR2A_0015*	chr17:7513454|7513675	-	Antisense	2.0303	222	CIRI2
*hsa-POLR2A_0016*	chr17:7514053|7514405	-	Antisense	2.0303	353	CIRI2
*hsa-POLR2A_0017*	chr17:7508944|7509632	+	Exonic	2.06061	348	find_circ, CIRI2,DCC,CIRCexplorer2
*hsa-POLR2A_0018*	chr17:7501036|7501383	+	Exonic	1.0303	168	CIRI2
*hsa-POLR2A_0019*	chr17:7500940|7501600	+	Exonic	2.0303	170	CIRI2
*hsa-POLR2A_0020*	chr17:7508944|7512188	+	Exonic	2.0303	871	CIRI2,DCC
*hsa-POLR2A_0021*	chr17:7497835|7499304	-	Antisense	1.0303	24	CIRI2
*hsa-POLR2A_0022*	chr17:7501588|7503175	-	Antisense	1.0303	604	CIRI2
*hsa-POLR2A_0023*	chr17:7501619|7502660	-	Antisense	2.0303	143	CIRI2
*hsa-POLR2A_0024*	chr17:7500782|7501576	-	Antisense	1.0303	51	CIRI2,DCC
*hsa-POLR2A_0025*	chr17:7501569|7501729	+	Exonic	1.0303	161	CIRI2,DCC
*hsa-POLR2A_0026*	chr17:7508251|7512923	+	Exonic	2.06061	150	find_circ, CIRI2,DCC,CIRCexplorer2
*hsa-POLR2A_0027*	chr17:7503466|7508327	-	Antisense	1.0303	102	CIRI2
*hsa-POLR2A_0028*	chr17:7484464|7496463	-	Antisense	1.0303	24	CIRI2
*hsa-POLR2A_0029*	chr17:7503466|7508347	-	Antisense	2.0303	122	CIRI2
*hsa-POLR2A_0030*	chr17:7503654|7508475	+	Exonic	1.0303	363	CIRI2,DCC
*hsa-POLR2A_0031*	chr17:7509532|7512590	+	Exonic	1.0303	101	find_circ, CIRI2,CIRCexplorer2
*hsa-POLR2A_0032*	chr17:7503121|7503490	+	Exonic	1.0303	172	find_circ, CIRI2,DCC,CIRCexplorer2
*hsa-POLR2A_0033*	chr17:7502026|7502660	-	Antisense	1.0303	240	CIRI2
*hsa-POLR2A_0034*	chr17:7499146|7499482	-	Antisense	1.0303	202	CIRI2
*hsa-POLR2A_0035*	chr17:7500560|7501095	-	Antisense	1.0303	445	CIRI2
*hsa-POLR2A_0036*	chr17:7503121|7512590	+	Exonic	1.0303	1850	find_circ, CIRI2,DCC,CIRCexplorer2
*hsa-POLR2A_0037*	chr17:7513664|7513833	-	Antisense	1.0303	170	CIRI2
*hsa-POLR2A_0038*	chr17:7509532|7511319	+	Exonic	1.0303	206	find_circ, CIRI2,DCC,CIRCexplorer2
*hsa-POLR2A_0039*	chr17:7513517|7513726	-	Antisense	3.0303	210	CIRI2
*hsa-POLR2A_0040*	chr17:7513732|7513943	-	Antisense	2.0303	212	CIRI2
*hsa-POLR2A_0041*	chr17:7513664|7513980	-	Antisense	3.0303	317	CIRI2
*hsa-POLR2A_0042*	chr17:7513876|7514093	-	Antisense	3.0303	218	CIRI2
*hsa-POLR2A_0043*	chr17:7513454|7513768	-	Antisense	2.0303	315	CIRI2
*hsa-POLR2A_0044*	chr17:7513652|7513821	+	Exonic	2.0303	170	CIRI2
*hsa-POLR2A_0045*	chr17:7513786|7513959	-	Antisense	2.0303	174	CIRI2
*hsa-POLR2A_0046*	chr17:7503466|7503683	-	Antisense	2.0303	123	CIRI2
*hsa-POLR2A_0047*	chr17:7513454|7513959	-	Antisense	2.0303	506	CIRI2
*hsa-POLR2A_0048*	chr17:7495941|7503791	+	Exonic	1.0303	Unknown	CIRI2
*hsa-POLR2A_0049*	chr17:7513597|7513756	-	Antisense	2.0303	160	CIRI2
*hsa-POLR2A_0050*	chr17:7513118|7513264	-	Antisense	2.0303	147	CIRI2
*hsa-POLR2A_0051*	chr17:7513454|7513596	-	Antisense	1.0303	143	CIRI2
*hsa-POLR2A_0052*	chr17:7513454|7513707	-	Antisense	2.0303	254	CIRI2
*hsa-POLR2A_0053*	chr17:7513828|7514009	-	Antisense	2.0303	182	CIRI2
*hsa-POLR2A_0054*	chr17:7513517|7513833	-	Antisense	3.0303	317	CIRI2
*hsa-POLR2A_0055*	chr17:7513454|7513714	-	Antisense	1.0303	261	CIRI2
*hsa-POLR2A_0056*	chr17:7513576|7513714	-	Antisense	2.0303	139	CIRI2
*hsa-POLR2A_0057*	chr17:7509043|7509632	+	Exonic	2.0303	249	CIRI2
*hsa-POLR2A_0058*	chr17:7496210|7496613	+	Exonic	2.0303	174	CIRI2
*hsa-POLR2A_0059*	chr17:7513906|7514082	-	Antisense	2.0303	177	CIRI2
*hsa-POLR2A_0060*	chr17:7512894|7513173	+	Exonic	1.0303	30	CIRI2
*hsa-POLR2A_0061*	chr17:7497853|7498192	-	Antisense	1.0303	115	CIRI2
*hsa-POLR2A_0062*	chr17:7503091|7503402	-	Antisense	1.0303	220	CIRI2
*hsa-POLR2A_0063*	chr17:7513669|7513997	-	Antisense	2.0303	329	CIRI2
*hsa-POLR2A_0064*	chr17:7513918|7514052	-	Antisense	1.0303	135	CIRI2
*hsa-POLR2A_0065*	chr17:7498936|7504215	+	Exonic	1.0303	Unknown	CIRI2
*hsa-POLR2A_0066*	chr17:7488471|7491379	+	Non-repeat	1.0303	Unknown	CIRI2
*hsa-POLR2A_0067*	chr17:7496443|7496581	-	Antisense	1.0303	139	CIRI2
*hsa-POLR2A_0068*	chr17:7513664|7513875	-	Antisense	2.0303	212	CIRI2
*hsa-POLR2A_0069*	chr17:7484733|7495962	+	Exonic	1.0303	147	CIRI2
*hsa-POLR2A_0070*	chr17:7513669|7513850	-	Antisense	2.0303	182	CIRI2
*hsa-POLR2A_0071*	chr17:7506353|7506510	+	Non-repeat	1.0303	158	CIRI2
*hsa-POLR2A_0072*	chr17:7512551|7512923	+	Exonic	1.0303	190	CIRI2
*hsa-POLR2A_0073*	chr17:7484696|7496036	+	Exonic	1.0303	258	CIRI2
*hsa-POLR2A_0074*	chr17:7513664|7513835	-	Antisense	1.0303	172	CIRI2
*hsa-POLR2A_0075*	chr17:7509532|7511588	+	Exonic	1.0303	101	CIRI2
*hsa-POLR2A_0076*	chr17:7498078|7503791	+	Exonic	1.0303	Unknown	CIRI2
*hsa-POLR2A_0077*	chr17:7503121|7512923	+	Exonic	1.0303	156	CIRI2

CircRNAs can also modulate *POLR2A* activity themselves. In ovarian cancer, circ*METTL6* has been identified as a tumor suppressor, where its overexpression inhibits metastatic growth and prolongs survival (Yu et al., 2025). Mechanistic studies revealed that loss of circ*METTL6* increases NONO expression and stabilizes RPB1, promoting GDF15 transcription and metastatic potential (Yu et al., 2025). These results suggest that circRNAs derived from other genomic loci can influence *POLR2A*/RPB1-dependent transcriptional programs in cancer.

Regulation of *POLR2A* may also involve other classes of ncRNAs, including miRNAs. miRNAs primarily control gene expression by binding to target mRNAs in the cytoplasm, which can result in mRNA degradation and subsequent recycling of its components or in preserving the mRNA for later translation (O’Brien et al., 2018). Depending on the context, their dysregulation can contribute to tumorigenesis, acting as oncogenes or tumor suppressors. Altered miRNA expression profiles have been linked to key processes such as regulation of molecular landscape, proliferation, apoptosis, and metastasis, making them valuable biomarkers for cancer diagnosis and prognosis (Rooj et al., 2017; Metcalf, 2024). Furthermore, therapeutic strategies targeting miRNAs are being explored, offering promising avenues for personalized cancer treatment (Kim and Croce, 2023). The role for miR-378a-3p was discovered in sarcomatoid renal cell carcinoma (sRCC) using clinically derived cell lines. The authors showed that the mRNA and protein expression levels of *POLR2A* and *RUNX2* were significantly higher than in other RCC subtypes. Mechanistically, increased levels of miR-378a-3p expression in sRCC cell lines (RCC52) suppressed RPB1 and RUNX2 and enhanced apoptosis (Weng et al., 2022). These findings highlight the possible intricate post-transcriptional regulation of *POLR2A* by a diverse array of miRNAs, underscoring their potential as modulators of oncogenic pathways.

## 8 Conclusion and future directions

Recent results have underscored the pivotal role of *POLR2A*/RPB1 dysregulation in driving tumor growth and progression across various cancer types. Elevated *POLR2A* expression is consistently associated with increased cell proliferation, altered cell-cycle dynamics, and resistance to apoptosis, highlighting its importance in sustaining oncogenic programs. Moreover, studies suggest that *POLR2A*/RPB1 interacts with various proteins to form regulatory networks that may drive aggressive tumor behavior and affect treatment responses.

Future efforts should focus on dissecting the complex molecular crosstalk between *POLR2A* and key cellular regulators. Integrative approaches, combining multi-omics profiling, functional genomics, epitranscriptomics, and advanced computational modeling, will be crucial for fully mapping these networks and identifying new therapeutic vulnerabilities.

The promising anticancer potential of natural compounds warrants further exploration as they may offer innovative strategies to modulate *POLR2A*/RPB1 activity and combat drug resistance. Bioactive compounds such as triptolide have already demonstrated the ability to disrupt transcriptional machinery. Future research should focus on elucidating the precise molecular mechanisms by which these compounds regulate *POLR2A* and its associated pathways, potentially unveiling new therapeutic targets. Moreover, pairing natural compounds with advanced drug-delivery platforms, including nanoparticle-based systems, may enhance their stability, specificity, and clinical efficacy.

The recurrent targeting of *POLR2A*/RPB1 by phylogenetically diverse viruses further highlights its role as a nonredundant bottleneck within the cellular transcriptional apparatus. Through mechanisms such as cap-snatching, CTD phosphorylation rewiring, and proteasome-mediated RPB1 degradation, viruses exploit Pol II to silence host transcription and enforce viral gene expression. The CTD code, once viewed as rigid, is now understood as a dynamic regulatory interface that viruses can reprogram to establish noncanonical transcriptional states. Intriguingly, similar principles appear to operate in cancer, where *POLR2A*/RPB1 dysregulation fuels transcriptional addiction and malignant progression. These parallels suggest a unifying conceptual framework: understanding RPB1 vulnerabilities may inspire therapeutic innovation spanning transcription-targeted agents to rationally engineered oncolytic viruses. Although still speculative, viruses such as HSV-1 could potentially be modified to exploit Pol II activity selectively within tumors, dismantling cancer-specific transcriptional programs while amplifying viral oncolysis.

Another emerging dimension of *POLR2A*-driven oncogenesis is the production of circRNAs derived from the *POLR2A* transcript. CircRNAs are increasingly recognized as important regulators of gene expression and cancer biology. Continued research into the circularization of the pre-*POLR2A* transcript may clarify how these molecules contribute to tumor progression and could lead to the development of new diagnostic tools and therapeutic strategies. Because *POLR2A* is a protein-coding gene, identifying potential open reading frames within circ*POLR2A* isoforms may also shed light on noncanonical translation events in cancer. High-throughput sequencing will be essential for cataloging the full repertoire of circ*POLR2A*s, ultimately enabling the discovery of novel biomarkers and actionable therapeutic targets.
